# Impact of food processing on rye product properties and their in vitro digestion

**DOI:** 10.1007/s00394-017-1450-y

**Published:** 2017-04-17

**Authors:** Daniel P. Johansson, José L. Vázquez Gutiérrez, Rikard Landberg, Marie Alminger, Maud Langton

**Affiliations:** 1 Department of Molecular Sciences, Swedish University of Agricultural Sciences (SLU), Uppsala, Sweden; 2 Nutritional Epidemiology Unit, Institute of Environmental Medicine, Karolinska Insitutet, Stockholm, Sweden; 3 Department of Biology and Biological Engineering, Food and Nutrition Science, Chalmers University of Technology, Gothenburg, Sweden

**Keywords:** In vitro digestion, Glucose release, Microstructure, Dietary fibre, Gastric digestion, Rye

## Abstract

**Purpose:**

Rye products have been reported to elicit postprandial insulin and glucose responses which may be beneficial for prevention of type-2 diabetes. However, mechanisms underlying variations in responses related to processing techniques are not fully understood.

**Methods:**

Five differently processed rye products (sourdough-fermented bread, fermented and unfermented crispbread, extrusion-cooked rye, and porridge) and refined wheat bread were characterised. Two in vitro methods, a dynamic method simulating digestion in the stomach and small intestine and a static method, simulating conditions in the stomach were used to determine viscosity development, structural changes and release of glucose during digestion.

**Results:**

Structural and compositional differences induced by processing influenced product digestion. Gastric disintegration and digesta particle size were related to characteristics of the starch/protein matrix, while digesta viscosity was reduced due to fibre degradation during fermentation. More cohesive boluses were associated with slower glucose release. Sourdough fermentation increased amylose leakage and appeared to inhibit starch hydrolysis despite low digesta viscosity and rapid disintegration.

**Conclusions:**

The net release of glucose during digestion of foods is determined by several factors which may vary in their importance depending on product specific properties.

## Introduction

High postprandial glucose and insulin concentrations in the blood may induce oxidative stress and low-grade inflammation [1], and result in decreased insulin sensitivity and subsequent increased risk of developing type-2 diabetes [2]. Foods which elicit beneficial glucose and hormonal responses may, therefore, help prevent cardiovascular diseases and type-2 diabetes.

Rye (Secale cereal L.) is an important crop in Eastern, Central and Northern Europe and a rich source of dietary fibre [3]. In acute intervention studies on healthy subjects, rye products have repeatedly been shown to induce lower postprandial insulin response, with or without a corresponding decrease in glucose response, compared with refined wheat bread [4–7]. The underlying mechanisms are not known, but have been suggested to relate to structural features of rye products, such as dense structure and formation of an amylose layer around starch granules, inhibiting starch hydrolysis [4]. In addition, high content and molecular weight of certain fibres in rye could influence glucose absorption rate and hormonal responses through decreased diffusion rates of nutrients [8, 9]. In a recent study, unfermented whole grain rye crispbread was observed to induce a lower insulin response than a corresponding fermented product [5]. This was attributed partly to degradation of β-glucan and arabinoxylan by endogenous enzymes during fermentation.

Other processes and processing conditions may also affect starch hydrolysis and postprandial glucose and insulin responses to rye products, but comparative studies are lacking. In cereal-based foods, botanical integrity, e.g., cracked grains compared with flour, decreases the release rate of nutrients by limiting access by enzymes [10]. Extrusion cooking with high temperatures and mechanical forces results in loss of structural integrity and a high degree of starch gelatinisation, which can increase in vitro digestibility of starches [11]. Water availability, cooking time and temperature also affect starch gelatinisation, with implications for the rate and extent of hydrolysis [12]. Moreover, dense food structure may decrease the rate of starch hydrolysis and the postprandial glucose and insulin responses, as shown for pasta compared with white bread [13]. Furthermore, while fibre degradation during fermentation may increase the rate of starch hydrolysis and absorption, organic acids produced during sourdough fermentation may counteract this through inhibition of starch hydrolysis and reduced gastric emptying [14].

Although human studies are usually considered the most reliable approach for accurate prediction of release and absorption of nutrients through the gut wall, establishment of mechanistic relationships is complicated. For example, the concentration of glucose in circulation is not a direct reflection of absorption, as it also depends on endogenous production rate and clearance rate [15], which are regulated by both insulin and the incretins GLP-1 and GIP, released in response to nutrient absorption [16]. In vitro methods (static or dynamic) are appropriate for mechanistic studies and widely used to predict the behaviour of food components in the digestive tract. Release of maltose and microstructural changes in food products during digestion has been studied previously using the dynamic in vitro model TNO Gastro-Intestinal Model (TIM) [17].

Most studies to date have focused on one product type, e.g., bread or extruded cereals, and how specific processing parameters or different raw materials affect in vitro digestion and postprandial responses in humans. Although different product types based on the same raw material have been compared, understanding of how differences in their properties and how they are digested and may contribute to postprandial responses, is limited. The aim of this study was to investigate how processing influences the microstructure and composition of rye products, the digesta properties and release of glucose during in vitro digestion. Five differently processed rye products, based on the same raw material, but differing in microstructure regarding the protein/starch matrix and the distribution and integrity of dietary fibre were therefore selected and compared with refined wheat bread. The results may also be of relevance when considering release of other compounds in cereal-based foods.

## Materials and methods

### Products

Of the six products included in the study, three were commercially available: refined wheat bread (WB) (Pågen AB, Sweden), yeast-fermented whole grain rye crispbread (RCB) and unfermented whole grain rye crispbread (uRCB) (Barilla Sweden AB, Sweden). Flour of the same origin and composition, but milled to different particle sizes, was used for production of RCB and uRCB. According to data provided by the manufacturer, the uRCB flour had 30–42% particles <125 µm and 20–28% > 1040 µm, while the corresponding proportions in the RCB flour were 51–57% and 3–6%, respectively. RCB was fermented in two steps; first 120 min at 29 °C, followed by 35 min with an increase from 30 to 38 °C. For uRCB, flour was mixed with water at 12 °C and then whipped at 6 °C to incorporate air into the batter.

The other three products were produced in-house, using the same flour as for RCB. Sourdough-fermented whole grain rye bread (sRB) was prepared by mixing 5150 g whole grain rye flour, 35.9 g NaCl, 1300 g commercial sourdough (Jästbolaget AB, Sweden), 125 g fresh yeast and 4405 g H_2_O. The dough was mixed for 4 min at low speed and 4 min at high speed in a dough mixer (Varimixer, Charlotte, NC, USA), proofed for 30 min at room temperature, divided into 900 g portions and placed in baking tins, and then put in a proofing chamber at 38 °C and 80% relative humidity for 40 min. Baking (50 min) was initiated at 230 °C with 8 s of steam, and then immediately lowered to 190 °C.

Extruded whole grain rye (extR) was produced at VTT Technical Research Centre of Finland (Espoo, Finland). Whole grain flour, with addition of 0.8% NaCl, was extruded using a co-rotating twin-screw extruder APV MPF 19/25 with conveying and mixing elements (Baker Perkins Group Ltd, Peterborough, UK). The flour feed rate was 50 g/min, water addition 3.0 ml/min, screw speed 400 rpm and in-barrel residence time around 2 min. Die pressure was measured using a pressure transducer (Dynisco Ltd., UK) in the die plate. The temperature profile of the four heating blocks was: 140–100–80–60 °C (from die exit to feeding section). The parameters measured were: torque 76–86% (of maximum 100%), pressure at die 3.9–4.4 bar and temperature at die 124 °C. The expanded products were cut by the cutter blade (in front of the die exit) into breakfast cereal spheres (5–7 mm in diameter) and oven-dried at 80 °C for 15 min.

Whole grain rye porridge (RP) was prepared by mixing 64 g whole grain rye flour and 0.58 g NaCl with 200 g boiling water and stirring with a spoon for 2 min to ensure good mixing. The porridge was allowed to rest for 3 min before use.

Salt content in the rye products was equal and based on the content in the commercially produced rye crispbreads. WB had a higher content of salt (1.5% db.).

### Chemical analysis of products

Samples were milled with a cyclone sample mill (Retsch, Haan, Germany). Samples with high water content (WB, sRB, RP) were freeze-dried prior to milling. RP was prepared as described above and immediately frozen in liquid nitrogen before freeze-drying. Extractable and unextractable dietary fibre content and composition were analysed according to the Uppsala method [18]. Content of arabinoxylan and arabinogalactan was calculated assuming an arabinose/galactose ratio of 0.69 in extractable arabinogalactan [19]. β-glucan, fructan and resistant starch content was analysed using a K-BGLU kit [20], a K-FRUC kit [21], and a K-RSTAR kit [22], respectively (Megazyme, Bray, Ireland). Calcofluor average molecular weight of β-glucan (M_cf_) was analysed using size exclusion chromatography with fluorescence detection [23]. Crude fat was determined according to the method described in the Official Journal of the European Communities [24] and protein according to the Kjeldahl method (N × 6.25) [25]. Dry content was determined by drying the samples at 105 °C for 16 h according to AACC method 44-15A.

### In vitro digestion

#### Mastication

Mastication of samples for the in vitro trials was conducted by one subject according to the method used by Ballance et al. [26]. For use in the TIM model, all expectorated samples were collected. For the simulated gastric digestion, due to the small sample volume used, the first three expectorated boluses were discarded to allow stabilisation of salivary flow and mastication behaviour [27]. The sample mass collected for the in vitro trials was based on expected dry matter content of sample boluses, as determined in three replicates per product. Dry matter content of the boluses used for each experiment was also determined.

#### Static gastric digestion and viscosity

An adaptation of the standard method proposed within the COST action Infogest was used for static gastric in vitro digestion [28]. Boluses were collected as described above and water added to give the same dry content in all samples (approximately 25%). To the bolus/water mixture, simulated gastric fluid, prepared as described by Minekus et al. [28], was added to give a final ratio of 1:1 (bolus/water mixture:simulated gastric fluid, dry matter content approximately 12.5%) after adjustment of pH to 3. Using a total volume of 25 ml, gastric digestion was then performed in a Rapid Visco Analyser (RVA) (Newport Scientific Pvt. Ltd., Australia) at a rate of 120 rpm, temperature 37 °C and 2-h run time, to monitor the development of viscosity during gastric digestion. Viscosity data was collected over 1-s periods. A standard RVA paddle was used, but the cup was modified by addition of three vertically attached plastic baffles (1 mm × 1 mm × 50 mm) evenly distributed on the inside of the cup. The addition of baffles was required to facilitate mixing and prevent the bolus from being dragged by the paddle. This gave a minimum gap of approximately 1 mm between the paddle and the baffles (standard gap is 1.9 mm between paddle and cup wall). The final viscosity, i.e., after 120 min, was used for viscosity comparison. Sodium bicarbonate was added to the remaining sample to neutralise the pH and stop pepsin activity, and the samples were stored at 4 °C before particle size determination on the same day. All samples were run in triplicate. Order of samples and replicates was randomised.

#### Particle size distribution

Samples collected from static in vitro gastric digestion were passed through a series of sieves with decreasing mesh size (3150, 2000, 1000, 600, 425 and 250 µm). Each sieve was carefully rinsed for 30 s with cold water before removal from the sieve stack to reduce the number particles of sizes below the mesh size retained. Excess water was then removed from the fraction retained in each sieving step by filtration and dry matter content of the remaining sample was determined. Particle size fractions are reported as a percentage of total dry weight.

#### TIM 

**Fig. 1:**
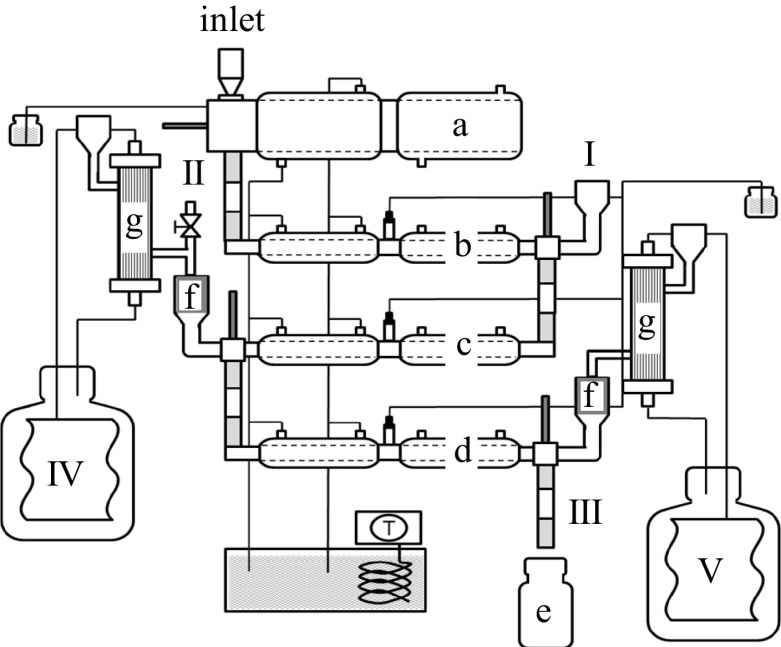
Schematic diagram of the TNO gastroi-intestinal model (*TIM*): **a** stomach, **b** duodenum, **c** jejunum, **d** ileum, **e** ileal delivery, **f** pre-filters, **g** dialysis filters. Roman numerals indicate sample collection sites: *I*) duodenum, *II*) jejunum, *III*) ileocaecal valve, *IV*) dialysis jejunum, *V*) dialysis ileum

The dynamic in vitro model TIM (TNO, Zeist, Netherlands) was used for simulating digestion in the stomach and small intestine (Fig. 1). This model consists of a series of compartments representing the stomach, duodenum, jejunum and ileum [29]. Solutions were prepared according to Salovaara et al. [30]. A medium meal transit time was chosen to simulate a semi-solid meal, and the half-time of stomach emptying was 70 min. The pH was measured every min and HCl was secreted into the gastric compartment to gradually decrease pH over time (pH/time (min): 6/0; 5.7/15; 4.5/45; 2.9/90; 2.3/120; 1.8/240; 1.6/300). NaHCO_3_ was secreted in the intestinal compartments to hold the pH on the set-point levels at approximately 6.5 in duodenum and 6.5-7-7.5 in jejunum and ileum. The same protocol was used for all products.

Mastication was performed as described above and boluses equal to a mass containing 20 g starch were collected. Water was added to give a total mass of 300 g (dry content 9–10%) and the product was fed into the stomach compartment of the model. The total time from end of mastication to initiation of in vitro digestion was 5 min. The in vitro digestion was performed in triplicate for WB, sRB and RP and in duplicate for RCB, uRCB and extR. Samples and replicates were run in randomised order.

Samples for glucose and maltose analysis were collected from the jejunum (II in Fig. 1) at 15, 30, 45, 60, 90, 120 and 180 min and boiled for 10 min to stop amylase activity. The samples were then immediately frozen at −20 °C and at the end of each day moved to a freezer at −80 °C for storage until analysis.

#### Glucose and maltose analysis

Prior to analysis, samples collected from the TIM model were thawed, centrifuged and diluted 200- to 2000-fold. Glucose and maltose concentrations were analysed using a Dionex HPAEC system (Dionex, Sunnyvale, CA, USA) consisting of a CarboPak PA-1 ion exchange column (4 × 250 mm with guard 4 × 50 mm) coupled with a pulsed amperometric detector. The mobile phase was run isocratically with a mixture of 95% 150 mM NaOH (A) and 5% 150 mM NaOH with 500 mM NaOAc (B) for 8 min for elution of glucose and maltose, and then increased to 100% B and maintained at 100% B for 6 min for elution of soluble carbohydrates of higher molecular weight. The mobile phase mixture was then reset to 95% A. The flow rate was 1 ml/min and total run time for each sample 20 min. Quality control samples run with each batch gave within-batch and between-batch variation of 1.4–3.6 and 4.4%, respectively. The sum of glucose and maltose recalculated to glucose was used for statistical analysis and is referred to as glucose in the text.

### Microscopy

Samples for microstructural characterisation were collected from the products, after mastication, after static simulated gastric digestion and in the TIM model from the duodenum (I in Fig. 1) at 30, 60, 90, 120 and 180 min, and from the ileal delivery (III in Fig. 1) at 60, 90, 120, 180, 240 and 360 min. All samples, except product samples to be embedded in plastic, were frozen in liquid nitrogen on collection and stored at −80 °C until analysis. Image J (fiji.sc/Fiji) was used for processing of micrographs and figures.

#### Immunolocalisation by confocal laser scanning microscopy (CLSM)

Frozen product samples were embedded in optimal cutting temperature compound and cut into 10 μm thick sections in a Leica CM1860 cryostat (Leica, Austria). The sections were incubated for 40 min in PBS buffer solution (0.1 M Na_2_HPO_4_, 0.1 M NaH_2_PO_4_, 0.15 M NaCl, pH 7.2) containing 1.5% bovine serum albumin (BSA) to prevent non-specific binding. The sections were then incubated for 2 h at 25 °C with primary antibody solution. Monoclonal antibodies raised against (1→3,1→4)-β-D-glucan (Biosupplies, Parkville, Australia) and arabinoxylan (LM11 antibody, Plant Probes, Leeds, UK) were diluted 1:100 and 1:50, respectively, in a PBS buffer solution containing 0.4% BSA. Appropriate controls were made by replacing the primary antibody solution with PBS buffer containing 0.4% BSA. After incubation, the sections were rinsed with PBS solution and incubated again for 40 min with PBS buffer solution containing 0.4% BSA. They were then incubated for 2 h in darkness with fluorescently labelled secondary antibodies, i.e. Alexa Fluor^®^ 488 goat anti-mouse IgG (H+L) and Alexa Fluor^®^ 568 goat anti-rat IgG (H+L) for BG and arabinoxylan, respectively, and finally rinsed with PBS and water. To achieve double immunolabelling, the whole procedure was carried out with anti-BG and Alexa Fluor^®^ 488 antibodies and then repeated on the same section with LM11 and Alexa Fluor^®^ 568 antibodies.

Micrographs were acquired using a Zeiss LSM 780 confocal microscope (Carl Zeiss, Germany) with a Plan-Apochromat 20×/0.8 M27 objective. A 488 nm Argon laser and a 561 nm diode pumped solid state laser were used as excitation sources and fluorescence emissions were collected between 493–578 nm and 570–640 nm. Zen2012 software (Carl Zeiss, Germany) was used in acquisition.

#### Bright field microscopy

Samples were fixed in 5% glutaraldehyde in 0.1 M phosphate buffer (pH 6.8) for 12 h, washed with phosphate buffer, fixed for another 2 h in 4% OsO_4_, and again washed in phosphate buffer. Samples were then dehydrated in a series of aqueous ethanol of increasing concentration, and finally infiltrated and polymerised with Technovit 7100. In vitro digested samples were embedded in cold gelling agar prior to the dehydration steps. To ensure representative samples of the masticated and digested samples, embeddings were made for each replicate and, in TIM, at each time point. Time points used for TIM were, after an initial screening, limited to: 60, 90 and 120 min from duodenum and 90, 120 and 180 from ileal delivery. A minimum volume of 0.5 ml of digesta was used for each embedding.

The embedded samples were cut into 1.5 µm thick sections with an ultra-microtome (Leica EM UC6, Leica, Austria) and stained with Lugol’s solution. The stained sections were examined using a Nikon Eclipse Ni-U microscope with 4×/0.20 and 40×/0.95 Plan-Apochromat objectives and images captured with a Nikon Digital Sight DS-Fi2 camera and processed with the software NIS-Elements BR (Nikon Instruments Europe, Amsterdam, Netherlands).

### Statistical analysis

Statistical analysis was conducted using SAS version 9.4 (SAS Institute Inc., Cary, NC, USA). Differences between glucose profiles were evaluated with a mixed effect model, PROC mixed, suitable for repeated measurements. For glucose profiles, separate models were used for concentration–time profiles and area under the curve (AUC). AUC was calculated using the trapezoid rule for the intervals between 0–90 and 0–180 min. For concentration models, time, product and a time × product interaction term were used as fixed effects, with time as a repeated variable. Run order was included as a random factor. When significant time × product interactions were found, pair-wise comparisons were performed at these time points. For AUC models, only product and run order were included in the model and pair-wise comparisons made. Differences in final viscosity between samples were evaluated using one-way ANOVA. All pair-wise comparisons were made using Tukey’s honest significance test.

## Results

### Chemical characterisation

Water content (% wb) was 35.7 and 53.0% for the soft breads WB and sRB, 7.5, 8.6 and 8.0% for the dry products RCB, uRCB and extR, and 77.0% for the semi-solid RP. The protein, fat and starch content were higher in WB than in the rye products, while the rye products contained more fibre (Table 1). Total fibre content was comparable between the rye products, but fibre composition varied. β-glucan and arabinoxylan differed only slightly in content, while average molecular weight (M_cf_) of β-glucan was lower in the fermented sRB and RCB than in the unfermented uRCB, extR and RP. β-glucan extractability was higher in the extruded rye product than in the other unfermented products. The extractable arabinoxylan content followed a similar pattern, being lowest in uRCB and RP. Resistant starch was highest in sRB and lowest in extR, but relatively similar in the other products.

**Table 1:** Product composition (%, dry basis) and calcofluor average molecular weight (M_cf_) of extractable β-glucan

	WB	sRB	RCB	uRCB	ExtR	RP
Protein	11.8	8.7	9.6	9.6	8.3	8.3
Fat	6.7	1.6	1.7	1.6	1.6	1.7
Available carbohydrates^a^	73.4	67.2	68.0	66.0	68.9	67.1
Resistant starch	1.8	3.3	0.8	0.7	0.1	1.2
Ash	2.2	2.3	2.4	2.3	2.3	2.2
Dietary fibre						
Total^b^	6.0	20.2	18.3	20.5	18.9	20.7
Extractable^c^	1.8	6.8	7.2	7.9	8.7	7.6
Unextractable	4.2	13.4	11.1	12.6	10.2	13.1
Arabinoxylan^d^						
Total	1.9	8.5	8.6	8.8	8.4	8.7
Extractable	0.9	3.0	3.0	2.6	3.4	2.5
Unextractable	1.0	5.6	5.6	6.3	5.1	6.2
Arabinogalactan	0.2	0.2	0.2	0.1	0.1	0.2
β-glucan	0.3	2.1	2.1	2.5	2.1	2.1
Cellulose and resistant starch^e^	2.4	4.6	2.5	2.7	2.1	3.2
Fructan	0.4	2.5	2.6	4.0	3.9	4.2
Klason lignin	0.1	1.5	1.3	1.3	1.3	1.5
β-glucan M_cf_ (10^5^ g/mol)	1.5	2.5	1.4	4.5	6.5	7.3
Extractability, β-glucan (%)	17	27	27	18	36	16

*WB* refined wheat bread. *sRB* sourdough-fermented whole grain rye bread. *RCB* yeast-fermented whole grain rye crispbread. *uRCB* unfermented whole grain rye crispbread. *extR* extruded whole grain rye. *RP* whole grain rye porridge

^a^Calculated by difference (total minus fat, protein, fibre and ash)

^b^Calculated as the sum of fructan and total dietary fibre, as analysed by the Uppsala method [18]

^c^Calculated as the sum of fructan and total extractable dietary fibre, as analysed by the Uppsala method [18]

^d^Calculated from the sum of arabinose, xylose and galactose, assuming an arabinose to extractable galactose ratio of 0.69 in arabinogalactan [19]

^e^Calculated as the difference between total β-glucan and glucose residues, as analysed by the Uppsala method [18]

### Product microstructure

**Fig. 2:**
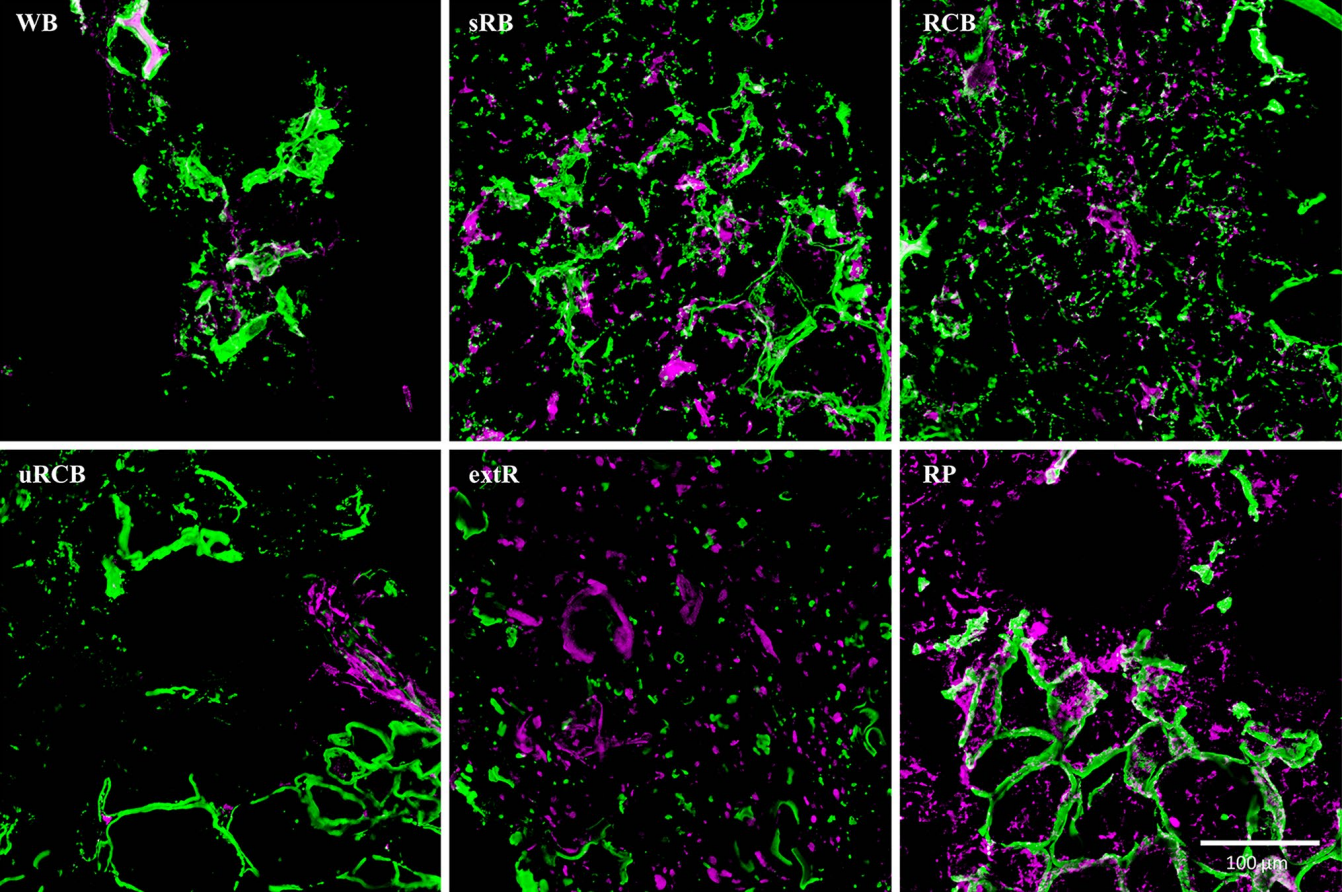
Confocal laser scanning microscopy micrographs of immunolabelled β-glucan (*green*) and arabinoxylan (*magenta*) in undigested products. *WB* refined wheat bread. *sRB* sourdough-fermented whole grain rye bread. *RCB* yeast-fermented whole grain rye crispbread. *uRCB* unfermented whole grain rye crispbread. *extR* extruded whole grain rye. *RP* whole grain rye porridge

Differences between the products in terms of structure and distribution of β-glucans and arabinoxylans were observed in the CLSM micrographs (Fig. 2). β-Glucan was distributed as smaller fragments throughout the matrix in the fermented WB, sRB and RCB, compared with the unfermented uRCB, extR and RP. Arabinoxylan followed a similar pattern, but was also distributed throughout the starch matrix in RP. In extR, β-glucan and arabinoxylan were clearly separated and distributed as smaller fragments throughout the matrix. The cell walls in RP appeared more swollen than in the other products.

**Fig. 3:**
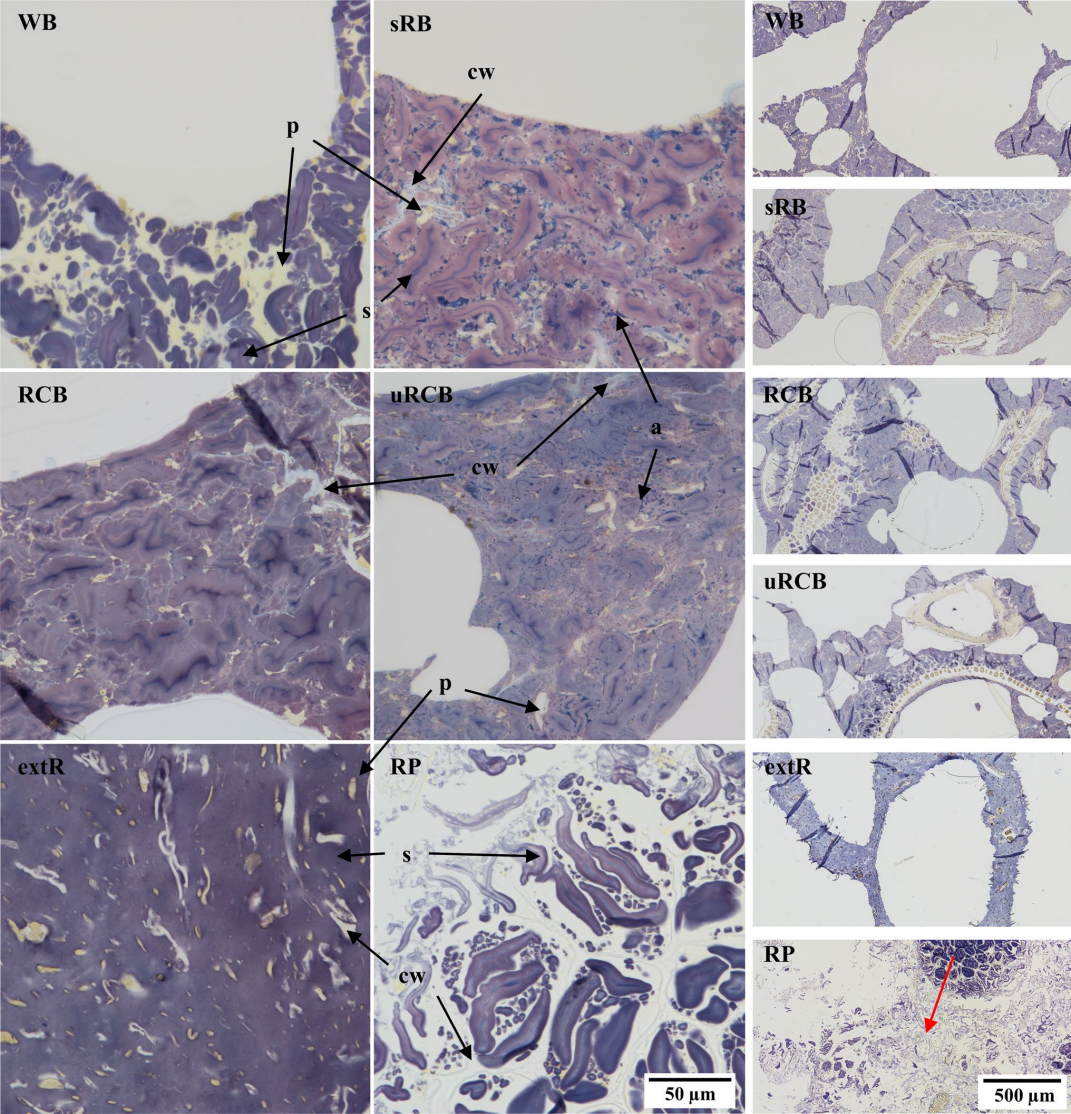
Light microscopy micrographs stained with iodine showing the microstructure of undigested products at two different magnifications. *WB* refined wheat bread. *sRB* sourdough-fermented whole grain rye bread. *RCB* yeast-fermented whole grain rye crispbread. *uRCB* unfermented whole grain rye crispbread. *extR* extruded whole grain rye. *RP* whole grain rye porridge. Protein (p) is *stained yellow*, *starch (s) purple*, amylopectin *brown* and amylose (**a**) *blue*. Cell walls (cw) are unstained, but can be seen in the starch/protein matrix. *Arrow* (**d**) indicates transition from intact structure to continuous dilute starch phase in RP

In WB, protein formed a continuous network, encapsulating starch granules, while all rye products had a continuous starch network encapsulating protein (Fig. 3). For RCB and uRCB, the starch phase consisted of highly swollen starch granules. In uRCB the granules were often indistinguishable, indicating a higher degree of starch gelatinisation than in RCB. The lamella also appeared thinner in uRCB than in RCB. Furthermore, uRCB contained larger pieces of bran and intact cell structures compared with the other rye products. In sRB, starch granules were less swollen and surrounded by a layer of leaked amylose. Leaked amylose was also observed in uRCB and, to a lesser extent, in RCB. In extR all starch granules were completely disrupted, resulting in a homogeneous starch phase, which encapsulated small fragments of cell walls and aleurone layers. The thickness of the lamella in extR was comparable to that in uRCB. In RP, larger fragments, consisting of aleurone layers and starch granules encapsulated in intact cells, were separated by a continuous phase consisting of loose starch granules in a dilute phase of leaked starch.

### Mastication and simulated gastric digestion

**Fig. 4:**
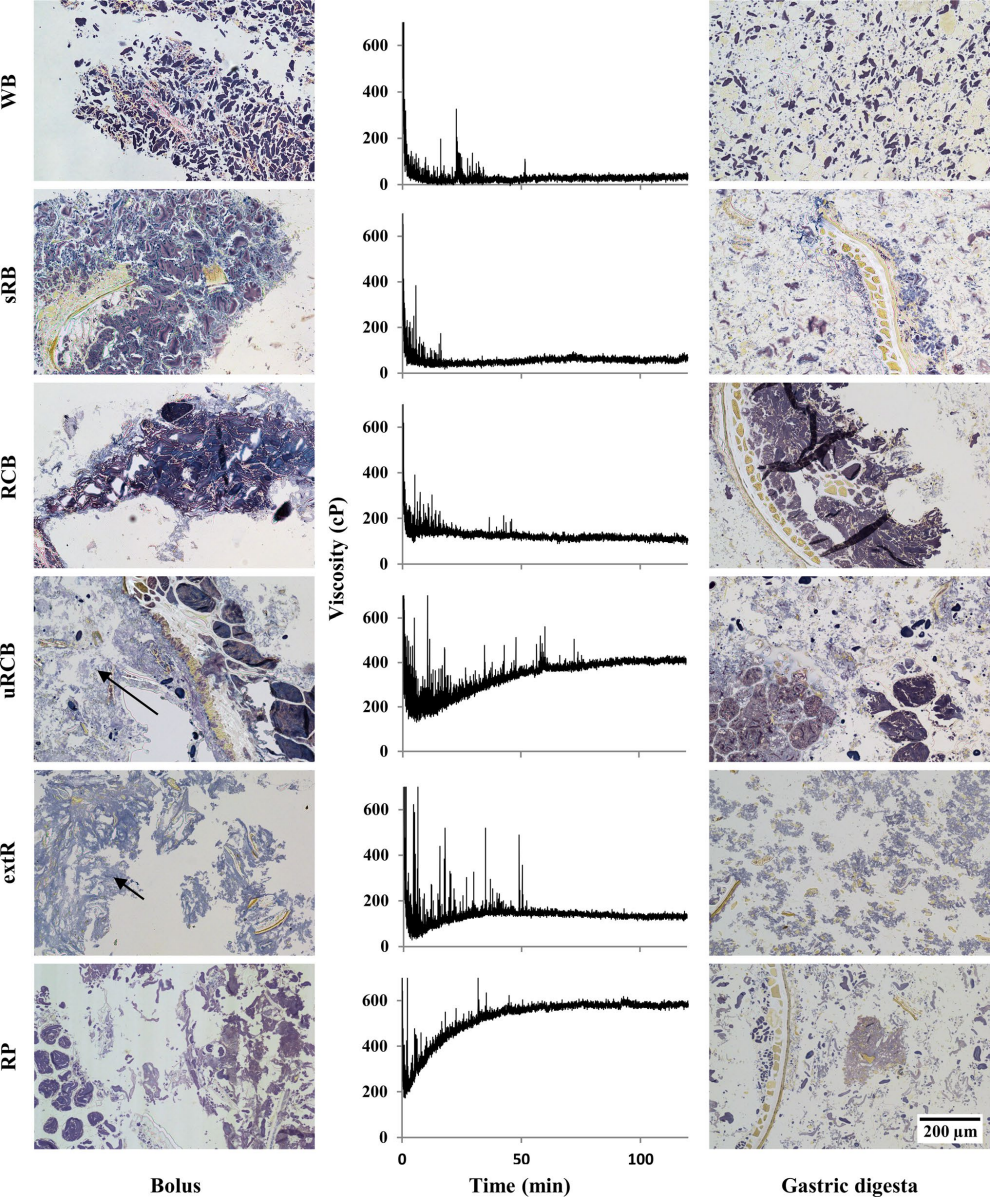
*Left* Light microscopy micrographs of the masticated samples. *Arrows* indicate the hydrated starch phase in uRCB and extR. *Centre* development of viscosity, measured by Rapid Visco Analyser at 120 rpm and 37 °C. *Right* LM micrographs, 120 min simulated gastric digestion. Protein is *stained yellow*, *starch purple*, *amylopectin brown* and *amylose blue*. *WB* refined wheat bread. *sRB* sourdough-fermented whole grain rye bread. *RCB* yeast-fermented whole grain rye crispbread. *uRCB* unfermented whole grain rye crispbread. *extR* extruded whole grain rye. *RP* whole grain rye porridge

The number of chewing cycles before expectoration varied between the products: 3–5 for RP, 10–15 for extR, 15–20 for WB, 20–25 for sRB and 25–30 for RCB and uRCB. Bolus water content (%wb, standard deviation in brackets) was 51.6% (2.0), 49.2% (2.2), 48.9% (1.9) and 47.2% (1.5) for WB, RCB, uRCB and extR, respectively, 60.5% (1.3) for sRB and 75.1% (0.1) for RP. During mastication, the protein/starch matrix of WB appeared to be compacted, forming a bolus consisting of aggregates of starch granules and protein (Fig. 4). For sRB and RCB, the protein/starch matrix instead appeared to have fractured during mastication, resulting in fragments retaining structural features of the original product matrix. In sRB, the fragments appeared more fractured than in RCB. The uRCB bolus also contained fragments, but these mainly consisted of aleurone layers or endosperm cells connected by a weakly stained starch phase, most likely due to hydration. For extR, a similar hydrated starch phase mainly characterised the bolus and, as in WB, the structure appeared to have been compacted into aggregates. RP appeared unaffected by the masticatory process.

After completed gastric digestion, the protein network in WB was completely digested, leaving only free starch granules. For sRB the fragments from the bolus had been mostly disintegrated, while for RCB fragments similar to those observed in the bolus remained. In both uRCB and extR the hydrated starch phase had been disrupted, but the fragments with intact cells in uRCB remained. For RP the effect appeared to be mainly one of dilution.

#### Gastric digesta: viscosity

**Fig. 5:**
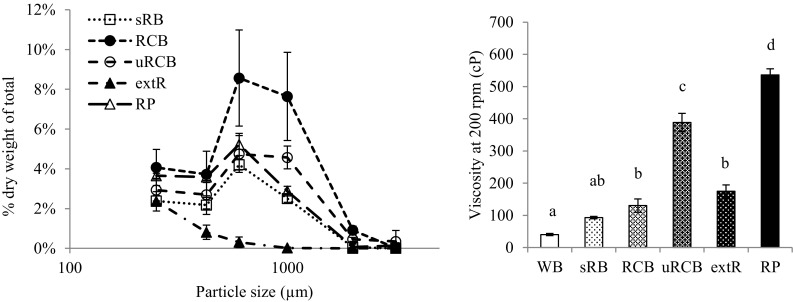
*Left* Particle size distribution as a percentage of dry weight of total (mean ± standard deviation) of particles after static gastric digestion. WB is not included in the graph as all particles were <250 µm in size. *Right* viscosity of digesta after 120 min (least square mean ± standard error) measured at 37 °C by Rapid Visco Analyser at 200 rpm. Different letters indicate statistically significant differences. *WB* refined wheat bread. *sRB* sourdough-fermented whole grain rye bread. *RCB* yeast-fermented whole grain rye crispbread. *uRCB* unfermented whole grain rye crispbread. *extR* extruded whole grain rye. *RP* whole grain rye porridge

The viscosity decreased rapidly within the first 5 minutes of simulated gastric digestion in the RVA for all products (Fig. 4). For WB, sRB and RCB, the viscosity then remained stable, while for uRCB, extR and RP it increased before stabilising. For all samples peaks could be observed in the viscosity curves. These were most likely caused by bolus fragments too large to pass between the paddle and the cup wall (i.e., > 2 mm) and consequently were caught between the paddle and the baffles giving rise to sudden and temporary increase in resistance to mixing. For uRCB and extR, the peaks occurred more frequently and for a longer time, 90 and 50 min, respectively, compared with the other products. The viscosity stabilised most rapidly for sRB, with no peaks observed after 20 min. The final viscosity was higher for uRCB and RP than for all other products and for extR and RCB compared with WB (Fig. 5).

#### Gastric digesta: particle size distribution

The WB digesta contained the smallest particles, with none above 250 µm (Fig. 5). Of the rye products, extR had the smallest particles (96% < 250 µm), followed by sRB, RP and uRCB (89, 84 and 83% < 250 µm, respectively). RCB contained the highest fraction large particles, with 75% below 250 µm.

### In vitro digestion of food products in the TIM model

**Fig. 6:**
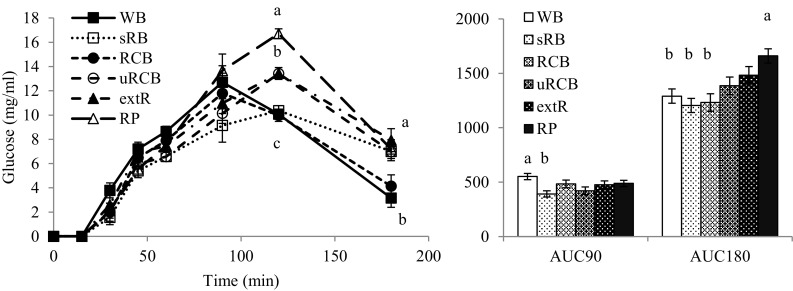
Glucose concentration profile and area under the curve (*AUC*). *Left* Glucose concentration**–**time profile (mean values). A statistically significant interaction between product × time was detected (*p* < 0.05). Different letters indicate statistically significant differences between products at certain time points (*p* < 0.05). *Right* Differences in total AUC_0–180min_ and AUC_0–90min_ between products. Values are adjusted least square mean ± standard error. *p* < 0.05 was considered significant. *WB* refined wheat bread. *sRB* sourdough-fermented whole grain rye bread. *RCB* yeast-fermented whole grain rye crispbread. *uRCB* unfermented whole grain rye crispbread. *extR* extruded whole grain rye. *RP* whole grain rye porridge

The glucose concentrations and AUC values differed between the products and there was a statistically significant product × time interaction (Fig. 6). The AUC value for the whole curve, AUC_0–180 min_, was lower for WB, sRB and RCB than for RP. For AUC_0–90 min_, only sRB was significantly lower than WB (*p* < 0.05). For specific time points, the glucose concentration at 120 min was higher for RP than for all other products and for uRCB and extR compared with WB, sRB and RCB. At 180 min, glucose concentration was higher for sRB, extR and RP compared with WB. For WB and RCB, the peak value occurred at 90 min, while for all other products it occurred at 120 min.

**Fig. 7:**
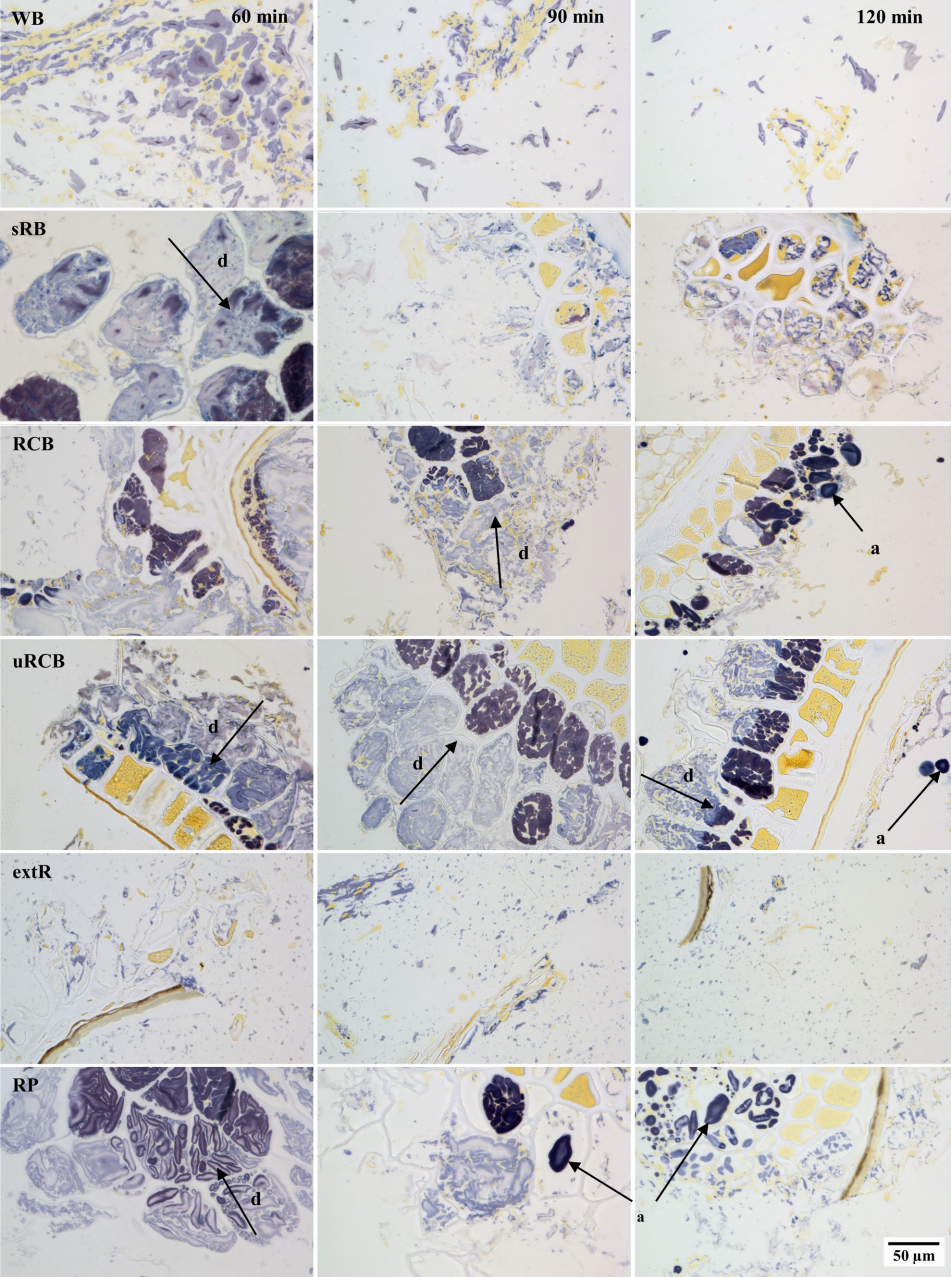
Light microscopy micrographs stained with iodine showing the microstructure of samples taken from the duodenal compartment after 60, 90 and 120 min digestion. Arrows marked with d indicate the direction of starch hydrolysis. Ungelatinised starch (**a**) was seen in RCB, uRCB and RP. *WB* refined wheat bread. *sRB* sourdough-fermented whole grain rye bread. *RCB* yeast-fermented whole grain rye crispbread. *uRCB* unfermented whole grain rye crispbread. *extR* extruded whole grain rye. *RP* whole grain rye porridge

Progressive degradation of the products was observed in samples collected from the duodenal compartment at different time points (Fig. 7). In the WB digesta, the protein/starch aggregates found in the bolus could be observed initially. Over time, these aggregates decreased in size and after 120 min were almost absent. In all rye products except extR, the digestion of starch was observed to proceed from the outside towards the centre of larger fragments. The amount of starch remaining also appeared to decrease with time. Fragments consisting of intact endosperm cells and aleurone layers were observed in sRB, RCB, uRCB and RP, while in RCB there were also fragments consisting of only starch granules, as observed after simulated gastric digestion by RVA. In extR, the digesta consisted of notably smaller starch particles than those seen after simulated gastric digestion. In general, the observations from the TIM model were in line with the particle size distribution after 2-h simulated gastric digestion. The main discrepancies were observed for WB, where larger aggregates were initially present, and extR, where the particles appeared smaller.

## Discussion

In the present work, we found differences in viscosity development, structural disintegration and glucose release during in vitro digestion of rye products depending on processing technique used. These results may contribute to the understanding of mechanisms underlying the beneficial postprandial responses observed for rye products [4, 6] and differences in postprandial responses observed for differently processed rye products [5, 7].

### Influence of product properties on mastication and simulated gastric digestion

The development of digesta viscosity for the different products will depend on the progressive disintegration of the bolus and hydration, swelling and solubilisation of different components [31]. The rapid initial decline in viscosity and the progressive decrease in frequency and amplitude of peaks was most likely the result of disintegration of the boluses. Although this does not give a direct measure of size or amount of larger fragments the longer duration of occurring peaks could indicate a slower disintegration of the boluses of uRCB and extR (Fig. 4). This could be related to more cohesive boluses resulting from the formation a connective starch phase during mastication, as observed for uRCB and extR (Fig. 4). The thinner lamella and more disrupted and gelatinised starch granules in uRCB and extR compared with sRB and RCB (Fig. 3) may have resulted in structures which were more easily hydrated by saliva during mastication. Plasticisation of the starch phase could give more flexible structures which were compacted during mastication, rather than fractured as appeared to be the case for sRB and RCB (Fig. 4). The continuous protein phase in WB may in a similar way have contributed to a more cohesive bolus compared with sRB and RCB. Refined wheat bread has also been reported to form larger particles after mastication than wholegrain and endosperm rye sourdough bread [32]. The viscosity curve for sRB seemed to stabilise rapidly compared with the other products, including RCB. This could be related to a weaker structure which was more easily disintegrated. Fractures were also observed in the bolus fragments (Fig. 4). The formation of fractures may have been promoted by less swollen, amylose-surrounded starch granules in sRB. sRB also appeared more disintegrated than RCB after completed gastric digestion (Fig. 4), as reflected in the particle size distribution (Fig. 5). Peaks in the viscosity curve were also observed for the semi-solid RP, indicating presence of larger bolus fragments. As RP was used in a heated state, submersion of the bolus in the colder simulated gastric fluid most likely resulted in gelling of the continuous starch phase and solidification of the bolus which then disintegrated.

In the present work only one individual was used to chew the products and expectorate prior to swallowing (and transfer into the stomach of the dynamic model), since previous investigations have reported that the inter-individual variability of food bolus particle size is very limited, as is the effect of salivary α-amylase in relation to the action of pancreatic α-amylase [26, 33, 34]. A limitation with mastication is that smaller particles may be lost by “intermediary swallowing” following dispersion in the saliva, and thereby not included in in vitro digestion [35]. Further studies are needed to evaluate and identify key parameters involved in cereal starch digestion and to confirm that the use of one individual to chew is representative.

The observed differences in final viscosity between the products (Fig. 5) may partially relate to fibre composition. Lower molecular weight of β-glucan (Table 1) and disrupted cell walls (Fig. 2), as observed in sRB and RCB compared with uRCB, extR and RP, is an indication of fibre degradation. β-glucan is known to be degraded by endogenous enzymes, which become active with the increased moisture content during fermentation [36]. Comparing uRCB with extR and RP, the time needed for mixing and baking of uRCB appeared sufficient for some degradation to occur. Although not analysed, molecular weight of arabinoxylans has been shown to be degraded similarly, but not to the same extent, as β-glucan [37, 39]. Arabinoxylan-degrading enzymes are mainly active at higher temperatures and lower pH, around 4.5, than β-glucanases [37, 39], and in uRCB, extR and RP arabinoxylan was probably relatively unaffected. In sRB and RCB; however, some degradation likely occurred. An increase in polydispersity and slight decrease in molecular weight has also been reported for fermented rye crispbreads compared with unfermented [38]. Furthermore, due to lower dough pH, the use of sourdough in sRB might have promoted more extensive degradation of arabinoxylan.

The digesta viscosity is also influenced by the characteristics, e.g., size and shape, of the particles present, although the relationship is not well known [31]. This would explain the relatively low digesta viscosity of extR, despite high extractability and retained molecular weight of β-glucan and high extractability of arabinoxylan, as it contained the smallest particles of the rye products (Fig. 5).

Viscosity and the rate of disintegration during gastric digestion can influence how rapidly starch becomes available for digestion in the small intestine. Solid foods are considered to be emptied from the gastric compartment first when reaching particle size < 1–2 mm [40]. Furthermore, in mixed meals, liquids are preferentially emptied first [41]. As boluses of cereal foods typically form more cohesive masses than e.g., vegetables [42], the rate of disintegration is likely to be of importance for gastric emptying and consequently the postprandial responses in humans [43]. High viscosity can also contribute to reduced gastric emptying [44]. Furthermore, both content of soluble fibres and viscosity can influence the diffusion rates of enzymes and glucose [8, 9, 45]. During digestion in vivo or in dynamic in vitro models, digesta viscosity will decrease due to continuous dilution by gastric and intestinal juices. However, differences in both viscosity and diffusion rates between products resulting from variations in particle characteristics and fibre composition may still persist.

### Influence of product properties on glucose release in the TIM model

The differences in glucose release between products were not clearly reflected in the progression of starch digestion as observed in the digesta from the TIM model at different time points (Fig. 7). The model has previously been used to visualise differences in starch digestion [17], but in that study a larger difference in total release of maltose was reported and both products tested, oat and barley tempeh, were relatively similar in structure. In our study a range of products with varying structures and properties were used, so different factors may have contributed to the concentration profiles of each product.

It is possible that slower gastric disintegration of uRCB and extR (Fig. 4) contributed to a later peak in glucose concentration, but similar AUC, compared with WB and RCB by decreasing the rate at which starch became available for digestion. No large bolus fragments (>2 mm) were observed in the intestinal compartments, and gastric sieving appears to occur in the TIM model too, indicating that gastric disintegration will be a factor to consider. Furthermore, lower diffusion rates due to less degraded fibres could decrease the rate of starch hydrolysis and, in the TIM model, reduce the rate of filtration/removal of digested compounds through the dialysis filters. This might also have contributed to the shift in glucose curves observed for uRCB and extR compared with WB and RCB. The highly disrupted starch in extR could have been expected to result in faster starch hydrolysis, but the gastric disintegration and diffusion rates may be more important factors. Despite similar glucose profiles in the TIM model, the responses to uRCB and extR may differ in humans, as the lower digesta viscosity of extR may result in faster gastric emptying rate [39]. The difference in glucose profiles between RCB and uRCB is in line with results of a recent comparison of RCB and uRCB with a refined wheat crispbread, where RCB produced an insulin response more similar to that of the wheat reference than to uRCB [5].

Differences in the glucose profiles for RCB and WB could have been expected considering the large differences in fibre content and composition, viscosity and particle size distribution. However, at equal viscosities, lower diffusion rates have been demonstrated for solutions of arabinoxylan from wheat compared with arabinoxylan from rye [9]. Moreover, as observed in the TIM model, particles were initially of comparable sizes for WB and RCB (Fig. 7). While starch hydrolysis rate is often reported to be higher for refined wheat breads than rye breads, measurements are usually made after simulated gastric digestion [4, 6] and the progressive changes in particle sizes occurring in vivo are not accounted for.

For RP, the large product volume, due to its high water content, may have contributed to its high glucose AUC. Compared with the other products, RP occupied a larger fraction of the volume in the gastric compartment and less liquid could be emptied before only the product remained, which should result in faster emptying of product. In humans this may compensate for slower gastric emptying due to high digesta viscosity [44]. Moreover, due to the high water content during preparation and the temperature of the product when initiating in vitro digestion, starch may have been more gelatinised and easily hydrolysed. The high AUC for RP compared with sRB is also in line with findings in a human study by Rosén et al. [7], where endosperm rye porridge and whole grain rye porridge induced higher glucose and insulin responses during the first 30 min than corresponding rye breads.

Neither of the explanations above can account for the low AUC_0–90min_ and late peak for sRB, with degraded fibres, low digesta viscosity and what appears to be the most rapidly disintegrated bolus of the rye products. However, the results are consistent with the lower insulin or lower insulin and glucose responses compared with refined wheat bread that have been repeatedly shown for soft rye breads, both sourdough-fermented and yeast-fermented [4, 6]. Although lactic acid produced during fermentation has been suggested to inhibit starch hydrolysis, with increased interaction between starch and gluten as the mechanism [46], the absence of a gluten network in sRB indicates other mechanisms. Rather, sRB was the only product where the presence of an amylose layer was observed, which has been suggested to inhibit starch hydrolysis in certain rye products [4]. The high content of resistant starch in sRB compared with the other products (Table 1) is likly related to the observed amylose layer (Fig. 3). To our knowledge, the factors contributing to the formation of an amylose layer in certain rye products are not known. However, organic acids produced during sourdough fermentation have been suggested to increase the content of resistant starch and may be a contributing factor [47]. Presence of an amylose layer may also be related to the high water content, compared with RCB, uRCB and extR, which could promote starch retrogradation during storage [48]. Whether the amylose layer is a result of sourdough fermentation or not warrants further investigation.

## Conclusions

From the results of this study, it is apparent that the processing technique affects the hydrolysis of starch and the release of glucose in rye products. Although highly gelatinised starch could be expected to increase the rate of glucose release, in dry products it seems to contribute to formation of a cohesive bolus during mastication which might lead to slower gastric disintegration and glucose release. The importance of different food structures for the bolus formation and the implications this has for the process of gastric disintegration however, need to be further investigated. Fermentation, with yeast or sourdough, leads to degradation of viscous fibres, which could contribute to faster diffusion and gastric emptying rates, increasing the release rate of glucose. However, in the case of rye sourdough bread it may also contribute to the formation of a protective amylose layer around starch granules instead of decreasing release rate. The large meal volume of porridge may contribute to more rapid emptying of product from the gastric compartment. Together with easily available starch, it may lead to faster starch hydrolysis despite high viscosity and intact fibres.

Overall, specific product properties, e.g., starch gelatinisation and fibre degradation, induced by food processing may affect the release rate of glucose in different directions and the net effect may differ between foods. This makes it difficult to draw conclusions on general effects of individual factors when comparing differently processed food products.
